# Rhizosphere Microbial Communities of *Spartina alterniflora* and *Juncus roemerianus* From Restored and Natural Tidal Marshes on Deer Island, Mississippi

**DOI:** 10.3389/fmicb.2018.03049

**Published:** 2018-12-11

**Authors:** Olga V. Mavrodi, Carina M. Jung, Jed O. Eberly, Samuel V. Hendry, Sanchirmaa Namjilsuren, Patrick D. Biber, Karl J. Indest, Dmitri V. Mavrodi

**Affiliations:** ^1^Department of Biological Sciences, The University of Southern Mississippi, Hattiesburg, MS, United States; ^2^U.S. Army Engineer Research and Development Center, Environmental Laboratory, Vicksburg, MS, United States; ^3^Gulf Coast Research Laboratory, The University of Southern Mississippi, Hattiesburg, MS, United States

**Keywords:** *Spartina altemiflora*, Juncus roemarianus, rhizosphere microbiome, coastal marshes, coastal restoration

## Abstract

The U. S. Gulf of Mexico is experiencing a dramatic increase in tidal marsh restoration actions, which involves planting coastal areas with smooth cordgrass (*Spartina alterniflora*) and black needlerush (*Juncus roemerianus*) for erosion control and to provide habitat for fish and wildlife. It can take decades for sedimentary cycles in restored marshes to approach reference conditions, and the contribution of the sediment microbial communities to these processes is poorly elucidated. In this study, we addressed this gap by comparing rhizosphere microbiomes of *S. alterniflora* and *J. roemerianus* from two restored marshes and a natural reference marsh located at Deer Island, MS. Our results revealed that plants from the restored and reference areas supported similar microbial diversity indicating the rapid colonization of planted grasses with indigenous soil microbiota. Although close in composition, the microbial communities from the three studied sites differed significantly in the relative abundance of specific taxa. The observed differences are likely driven by the host plant identity and properties of sediment material used for the creation of restored marshes. Some of the differentially distributed groups of bacteria include taxa involved in the cycling of carbon, nitrogen, and sulfur, and may influence the succession of vegetation at the restored sites to climax condition. We also demonstrated that plants from the restored and reference sites vary in the frequency of culturable rhizobacteria that exhibit traits commonly associated with the promotion of plant growth and suppression of phytopathogenic fungi. Our findings will contribute to the establishment of benchmarks for the assessment of the outcome of coastal restoration projects in the Gulf of Mexico and better define factors that affect the long-term resiliency of tidal marshes and their vulnerability to climate change.

## Introduction

Coastal marshes are highly productive ecosystems, exceeding primary production estimates of species rich ecosystems like tropical rainforests or coral reefs (Bertness, 2006). These wetlands provide key ecosystem benefits such as reducing fertilizer and pollutant run-off into the ocean, protection from storm surge, and habitat and shelter for many 100s of species of fish, birds, and other marine animals (Costanza et al., 1997). Despite the great faunal diversity, coastal marshes are dominated by a limited number of key species of perennial grasses, rushes, and sedges that have adapted to tolerate stresses associated with salinity changes, flooding, and temperature fluctuations. The most common of these plants in the northern Gulf of Mexico are black needlerush (*Juncus roemerianus*) and smooth cordgrass (*Spartina alterniflora*), which both form rhizomes and can appear in genetically identical tall and short forms (Eleuterius and McDaniel, 1978). The presence of these two marsh plant species represents a key component in the successful establishment and succession of coastal salt marshes. In the northern Gulf, marsh plant communities exhibit clear patterns of zonation, with *S. alterniflora* dominant in the low intertidal, and *J. roemerianus* dominant in the mid-marsh elevations, as a result of a host of biogeochemical factors (Craft et al., 2003; Herbert et al., 2015).

In Mississippi, coastal marshes have come under increasing pressure from both natural and anthropogenic sources. Increases in hurricane and storm activity combined with industrial and suburban development have resulted in significant habitat loss (Eleuterius, 1973). Such environmental issues can be best illustrated at Deer Island (GPS 30 21.99′–88 49.56′), which is located in the Mississippi Sound near the mouth of Biloxi Bay and the City of Biloxi, Mississippi. The 4.5-mile-long rod-shaped Deer Island originates from the late Pleistocene beach ridges and is not a true barrier island, but is a remnant of the mainland (Schmid and Otvos, 2003). Historical records indicate that Deer Island has suffered continuous erosion, resulting in the loss of approximately 300 acres over the last 150 years, or about one third of its 1850 footprint (Gerhardt Smith et al., 2015). While the island provides a diverse habitat for flora and fauna, certain portions of Deer Island’s shoreline are eroding rapidly, leading to a loss of sediment stabilizing vegetation. Increased exposure to wave energy, coupled with gradual sea level rise, has hastened erosion of wetlands along both Deer Island as well as the mainland of Mississippi. As a result, the island has been a pilot study site for wetland restoration by reestablishing *S. alterniflora* and *J. roemerianus* using beneficial use (BU) dredged material with the long-term goals of providing habitat for native species of the Gulf Coast and to aid the city of Biloxi, MS with protection during storm events (Gerhardt Smith et al., 2015).

**FIGURE 1:**
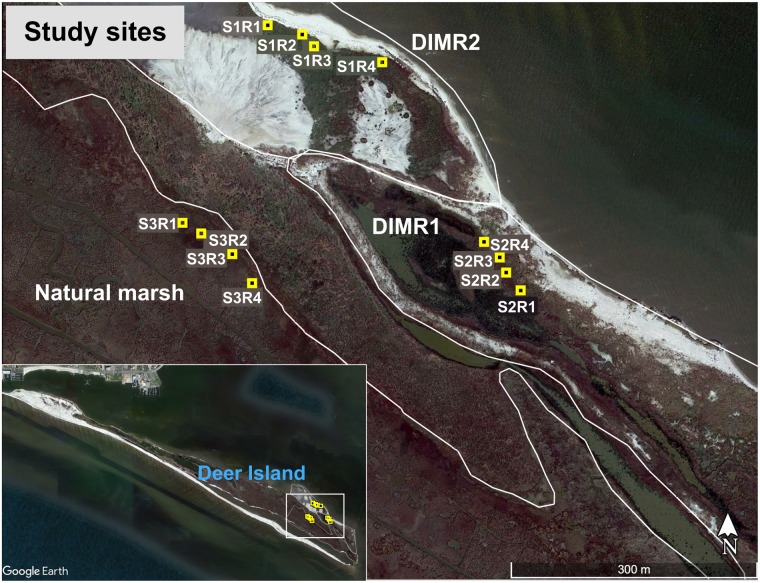
Map of Deer Island and the three surveyed and sampled areas.

The Mississippi Department of Marine Resources (MDMR) and the U.S. Army Corps of Engineers (USACE) began a multiyear restoration project of the eroded northeastern end of Deer Island in 2003 – Deer Island Multiyear Restoration (DIMR1) (Figure 1). Dredged material from the maintenance of the Biloxi Ship Channel was used to create approximately 30 acres of tidal marsh on the north shore of the east end of the island. After dewatering and stabilization of this fill, planting of *S. alterniflora*, *J. roemerianus*, and *Spartina patens* (salt marsh hay) along the sand-berm and selected areas of dredge fill was completed in spring 2005. Additional replanting of selected areas has been ongoing since then to stabilize the eroding sand-berm and new fill that has been added to the site since 2009 (Lang, 2012). A second round of berm construction and fill placement at DIMR1 was completed in 2013. In addition, a new phase of construction began in 2014 to approximately double the area of created marsh. This new area, DIMR2, was partially planted in spring of 2016 with approximately 36,000 plants of *S. alterniflora*, *J. roemerianus*, *S. patens*, *Uniola paniculata* (sea oats), and *Panicum amarum* (beach panic grass).

A post-construction assessment at Deer Island of the older constructed wetland (DIMR1) and an adjacent reference site showed the site dominated by *S. alterniflora*, *S. patens, Distichlis spicata* (salt grass), and *J. roemerianus* with no significant differences in abundance or diversity values across the sites (Lang, 2012). However, further monitoring of the newly constructed marsh site (DIMR2) at Deer Island, including more detailed microbial analysis of the sediments, is needed for a clearer understanding of the role of sedimentary ecosystem processes facilitated by the microbial community and the long-term stability of the site. Toward this objective we compared the rhizosphere microbial communities of the two dominant late successional plant species, *S. alterniflora* and *J. roemerianus*, from restoration sites DIMR1 and DIMR2 and a natural marsh reference site via high-throughput sequencing of 16S rRNA amplicons. We also isolated and characterized rhizobacteria that exhibit traits associated with the capacity to control phytopathogens and promote plant growth. In particular, we identified several species capable of antagonizing *Fusarium* spp., a group of plant pathogens which infect stressed *S. alterniflora* plants and play a significant secondary role in the ecological disruption of salt marshes during outbreaks of sudden vegetation dieback (SVD) (Elmer and Marra, 2011; Elmer, 2014). Collectively these findings reveal how microbial communities compare between natural marsh and restored sites as a function of construction practices and restoration age and help define potential factors that may affect the long-term restoration success and resiliency of coastal marshes.

## Materials and Methods

### Sample Collection and Processing

Three study sites, DIMR1, DIMR2, and natural marsh, were sampled over the course of this study (Figure 1). DIMR1 is a restoration site on the northeast of Deer Island, periodically planted between 2004 and 2015. DIMR2 was constructed to the west of DIMR1, with partial planting completed in 2015–2016. The natural marsh is a virgin, *Juncus*-dominated reference site located in the southeast portion of the island. The natural vegetation of the island exists in distinct elevation zones; with typical marsh zones dominated either by *S. alternifora* (low marsh), *J. roemerianus* (mid marsh), or a mixture of upland salt marsh vegetation including *S. patens, Baccharis halimifolia* (sea myrtle), and *D. spicata* (high marsh). At each site, the vegetation was surveyed by establishing a 100-m long transect perpendicular to the shoreline. The starting point of transect was within <3 m of a georeferenced permanent marker. Transects were surveyed on November 10, 2016, which represents the end of the growing season in the northern Gulf of Mexico and coincides with maximal live above-ground biomass resulting in decomposition and remineralization of the growing season organic biomass. Transect orientation was set to capture changes in plant zonation as a function of elevation change. Within each site and near the transect, several clumps each of *S. alterniflora* and *J. roemerianus* were selected randomly every few meters, dug with a shovel to below the sediment surface (about 30 cm) and placed in large plastic bags. Global positioning coordinates (GPSs) of collected plants from all sites were recorded (Table 1). Sediment samples (four per site) were collected from the same general locations using a 2′′ diameter soil core sampler (model no. 404.50, AMS, American Falls, ID, United States) for sediment geochemistry analysis on August 8, 2017. All collected plants and sediment cores were transported on ice to the laboratory at the Hattiesburg USM campus. In the laboratory, the plant material was kept at 4°C and processed within 24 h, while sediment samples were frozen at -20°C. Analysis of sediment samples was conducted by the Agricultural and Environmental Services Laboratories (AESL) at the University of Georgia (Athens, GA, United States) for total carbon and nitrogen, total organic carbon (TOC), ammonium, nitrate, nitrite, sulfate, and soil texture.

**Table 1:** Description of samples of *S. alterniflora* and *J. roemerianus* collected from the two DIMR restoration sites and natural marsh on Deer Island.

Site*^a^*	Plant species	Geographic coordinates ± 2 m	Sample group*^b^*
DIMR1	*S. alterniflora*	N 30°22.064′ W 088°49.456′	S2R1PX
DIMR1	*S. alterniflora*	N 30°22.076′ W 088°49.467′	S2R2PX
DIMR1	*S. alterniflora*	N 30°22.086′ W 88°49.472′	S2R3PX
DIMR1	*S. alterniflora*	N 30°22.096′ W 088°49.484′	S2R4PX
DIMR1	*J. roemerianus*	N 30°22.064′ W 088°49.456′	S2R1PX
DIMR1	*J. roemerianus*	N 30°22.076′ W 088°49.467′	S2R2PX
DIMR1	*J. roemerianus*	N 30°22.086′ W 88°49.472′	S2R3PX
DIMR1	*J. roemerianus*	N 30°22.096′ W 088°49.484′	S2R4PX
DIMR2	*S. alterniflora*	N 30°22.238′ W 088°49.648′	S1R1PX
DIMR2	*S. alterniflora*	N 30°22.232′ W 088°49.622′	S1R2PX
DIMR2	*S. alterniflora*	N 30°22.224′ W 088°49.613′	S1R3PX
DIMR2	*S. alterniflora*	N 30°22.214′ W 088°49.561′	S1R4PX
Natural marsh	*S. alterniflora*	N 30°22.102′ W 088°49.698′	S3R1PX
Natural marsh	*S. alterniflora*	N 30°22.108′ W 088°49.712′	S3R2PX
Natural marsh	*S. alterniflora*	N 30°22.088′ W 088°49.675′	S3R3PX
Natural marsh	*S. alterniflora*	N 30°22.069′ W 088°49.660′	S3R4PX
Natural marsh	*J. roemerianus*	N 30°22.102′ W 088°49.698′	S3R1PX
Natural marsh	*J. roemerianus*	N 30°22.108′ W 088°49.712′	S3R2PX
Natural marsh	*J. roemerianus*	N 30°22.088′ W 088°49.675′	S3R3PX
Natural marsh	*J. roemerianus*	N 30°22.069′ W 088°49.660′	S3R4PX

^a^*DIMR1 – an established site that was periodically planted through 2004–2015; DIMR2 – a newly developed site with partial planting done in 2015–2016; natural marsh – a reference site with native vegetation. Note absence of *J. roemerianus* from the DIMR2 site. ^b^The sample label indicates the site, replicate number, and plant number (e.g., S3R1P2 corresponds to the second *J. roemerianus* plant sampled from the natural marsh).*

### Isolation of Soil DNA and High-Throughput Sequencing of 16S rRNA Amplicons

To isolate soil DNA partial root systems were excised from larger clumps, excess sediment was carefully removed, and root systems with the adhering rhizosphere soil were placed into 50 mL Falcon tubes. Twenty milliliters of sterile saline were added to each tube and root-associated bacteria were dislodged by vortexing and treatment in a sonicating bath (1 min each). Rhizosphere soil DNA was extracted from 1.5 mL aliquots of root washes using a Power Soil DNA Isolation kit (MO BIO Laboratories, Carlsbad, CA, United States). The quality of the extracted DNA was verified by gel electrophoresis and PCR with primers 8F and 1492R that target eubacterial 16S rRNA (Weisburg et al., 1991). The amplifications were carried out in 50-μL reaction mixtures containing 1× DreamTaq DNA polymerase buffer, 200 μM each of dATP, dTTP, dGTP, and dCTP, 20 pmol of each primer, and 0.06 U of DreamTaq DNA polymerase (Thermo Fisher Scientific, Waltham, MA, United States). The amplifications were performed with a T100 gradient thermal cycler (Bio-Rad Laboratories, Hercules, CA, United States) and the cycling program consisting of initial denaturation at 94°C for 2 min followed by 30 cycles of 94°C for 20 s, 55°C for 15 s, and 72°C for 1.5 min, and a final extension at 72°C for 3 min. 16S rRNA amplicons for high-throughput sequencing were generated using the primer pair 515F-806R with each reverse primer containing a barcode following the protocol of Costello et al. (2009). Amplicons were then purified using a Promega Wizard PCR Cleanup kit (Madison, WI, United States), quantified, and sequenced on an Illumina MiSeq sequencer (Illumina, San Diego, CA, United States) using the Illumina MiSeq Reagent Kit v2 (300 cycles) following the manufacturer’s instructions. The 16S rRNA sequencing data from MiSeq runs were trimmed, demultiplexed and quality filtered using the default settings of the Illumina MiSeq Reporter software (v 2.6).

### Microbial Community Analysis

To compare bacterial microbiomes associated with roots of *S. alterniflora* and *J. roemerianus* collected at the three sites microbial community analysis was performed. From the 16S rRNA sequence data, OTU assignments were performed with the open reference OTU clustering package implemented in the QIIME (Quantitative Insights Into Microbial Ecology) bioinformatics pipeline (Caporaso et al., 2010) which performs an initial closed reference clustering against the Greengenes Database with a 97% sequence similarity threshold. Taxonomy assignments were performed using the naïve Bayesian Ribosomal Database Project (RDP) Classifier (Wang et al., 2007) as implemented in the MICCA (MICrobial Community Analysis) software pipeline (Albanese et al., 2015). Bar chart comparisons at different taxonomy levels were performed using a 1% relative abundance threshold to remove rare OTUs. Differences between sites were determined at the order level using a Kruskal–Wallis test with a significance threshold of *P* < 0.05. Order level was selected to provide the highest possible resolution of community composition between sites. Statistical comparisons of significant differences between individual phyla were performed using ANOVA. Analysis of similarities (ANOSIM) was performed using the compare_categories.py script in QIIME. Comparisons were made between *S. alterniflora* collected from DIMR1 and DIMR2 and natural marsh samples and between *J. roemerianus* collected from DIMR1 and natural marsh (this species was not found at DIMR2). Comparisons were also performed between *S. alterniflora* and *J. roemerianus* samples from the natural marsh site only to identify differences in community structure that were due to the type of vegetation present. Non-metric multidimensional scaling (NMDS) with Bray–Curtis distances was also performed in R Core Team (2014) using the Phyloseq package (Mcmurdie and Holmes, 2013). Samples were first filtered to remove rare OTUs (appearing less than five times in at least 50% of the samples). Following the filtering step, a variance-stabilizing transformation (VST) was applied. VST is a transformation method which removes the dependence of the variance on the mean (Anders and Huber, 2010). The resulting data set was pruned to the nine most abundant phyla for generating the NMDS plot.

### Isolation of Culturable Bacteria and Estimation of Bacterial Populations

Population levels of total culturable heterotrophic bacteria in the rhizosphere of *Spartina* and *Juncus* were estimated using the terminal dilution endpoint assay (Mavrodi et al., 2012). Root washes were serially diluted in microplates and cultured in one-third-strength King’s Medium B (^1^/_3_ KMB) (King et al., 1954) and 1-10th-strength Tryptic Soy broth (^1^/_10_ TSB) (Martin, 1975) supplemented with 15% artificial saltwater SW30 (Dyall-Smith, 2009) and 100 μg mL^-1^ of cycloheximide. The bacterial growth was estimated after 72 h of growth at 27°C by measuring optical density at 600 nm (OD_600_) with a Synergy 2 microplate reader (BioTek Instruments, Winooski, VT, United States). An OD_600_ of >0.1 was considered positive for bacterial growth. Population densities of bacteria were calculated based on the final dilution with positive growth. The detection limit of this technique was log 3.2 CFU g^-1^ root fresh weight. We also assembled a collection of rhizobacterial isolates by spread-plating the root washes on ^1^/_3_ KMB agar and 1-10th-strength Tryptic Soy agar (^1^/_10_ TSA) amended with 15% SW30 and cycloheximide to suppress fungi. The inoculated plates were incubated at 27°C for 72 h and distinct bacterial morphotypes were transferred onto fresh ^1^/_3_ KMB or ^1^/_10_ TSA and serially passaged to obtain pure cultures. All isolates were stored at -80°C in ^1^/_3_ KMB or ^1^/_10_ TSB supplemented with 30% glycerol for later screening. Bacterial population densities were converted to log CFU g^-1^ of fresh root weight. Differences among treatments in *S. alterniflora* were determined by the standard one-way analysis of variance (ANOVA) with mean comparisons among treatments performed by the Fisher’s protected least significant difference (LSD) test (*P* = 0.05) or by the Kruskal–Wallis test (*P* = 0.05). Bacterial populations in *J. roemerianus* were compared by the two-sample *t*-test (*P* = 0.05) or Wilcoxon Rank Sum test (*P* = 0.05). All statistical analyses were conducted with Statistix 10 (Analytical Software, Saint Paul, MN, United States).

### Screening of Rhizosphere Isolates for Plant Beneficial Traits

A subset of the rhizosphere isolate collection was screened for traits commonly associated with the capacity to proliferate in the rhizosphere (siderophores, motility), antagonize plant pathogens (production of exoprotease, biosurfactants, and HCN), and promote plant growth [production of ACC deaminase and indoleacetic acid (IAA)]. A total of 287 isolates (*n* = 55–61 per sampled plant species per site) from roots of *Spartina* and *Juncus* were randomly selected and cultured for 2 days at 27°C in 96-well microplates filled with ^1^/_5_ TSB supplemented with SW30. These microplates served as a source of inoculum for individual assays and allowed for the rapid transfer of bacteria with the help of a 96-pin replicator. The secretion of protease was assessed by observing a clearing zone surrounding bacterial growth on skim milk agar after 3 days of incubation at 27°C (Sacherer et al., 1994). The siderophore production was determined by pre-growing bacteria on ^1^/_5_ TSA with SW30 for 2 days at 27°C and then overlaying plates with 30 mL of soft O-CAS agar (Perez-Miranda et al., 2007). The overlayed plates were incubated at 27°C and the appearance of yellow/orange halo around bacterial colonies was scored at 4, 8, and 24 h. The production of hydrogen cyanide was monitored by spotting bacteria on TSA supplemented with 4.4 g L^-1^ glycine (Bakker and Schippers, 1987). The inoculated Petri dishes were sealed with Parafilm, and the production of HCN was detected by placing on the lid a piece of indicator filter paper impregnated with 0.5% picric acid and 2.0% sodium carbonate. The change in the color of the indicator paper from yellow to bright orange after 2–5 days of growth at 27°C indicated the production of HCN. The production of biosurfactants was assayed by the droplet collapse assay via spotting 10 μL of a culture grown in ^1^/_5_ TSB with SW30 onto a hydrophobic surface (Parafilm). The presence of biosurfactants in the culture medium causes the collapse of the droplet due to the reduction in the surface tension. The motility of bacteria was assessed by spotting bacteria on the semi-soft ^1^/_5_ TSA and scoring the zone of spread after the first and second day of incubation at 27°C. The growth on ACC as a sole nitrogen source was determined by a modified protocol of Penrose and Glick (2003). The bacteria were pre-grown for 2 days with shaking (190 rpm) at 27°C in deep-well microtiter plates filled with ^1^/_5_ TSB with SW30. The cells were washed twice and replica-plated onto DF agar without N source (control), or the DF agar supplemented with (NH_4_)_2_SO_4_ or 3.0 mM ACC. Plates were scored for growth after 2–4 days of incubation at 27°C. All isolates that were positive for growth on ACC were confirmed for the capacity to use this compound as a N-source in liquid DF medium. The production of IAA was determined by growing isolates at 27°C and 190 rpm in deep-well microplates filled with ^1^/_5_ TSB + SW30 with and without tryptophan (500 μg mL^-1^). After 2 days of incubation, the optical density at 600 nm of bacteria grown in two media was measured, and cells were removed by centrifugation at 5000 rpm for 10 min at 10°C. The presence of IAA was detected by mixing 40 μL of supernatant with 160 μL of Salkowski’s reagent (0.5 M FeCl_3_ and 35% perchloric acid) and measuring the absorbance at 540 nm after the 25-min incubation at room temperature (Gordon and Weber, 1951). All assays were repeated at least twice with two replicates per experimental condition. Frequencies of rhizobacteria with beneficial traits at DIMR1, DIMR2, and the natural marsh were compared using chi-square test (*P* = 0.05) with Statistix 10.

### Antagonism of Rhizosphere Isolates Toward Plant-Pathogenic *Fusarium*

The capacity of rhizosphere isolates to antagonize the growth of *Fusarium pseudograminearum* and *F. culmorum* was assayed on ^1^/_5_ TSA. A 5-mm-diameter agar plug of a 5-day-old culture of *Fusarium* grown on potato dextrose agar was transferred to the center of a ^1^/_5_ TSA plate. Next, 10 μL of an exponentially growing bacterial culture adjusted to an OD_600_ of 0.1 was spotted 1 cm from the edge the agar plate. The inoculated plates were incubated in the dark at 27°C for 5 days, after which the distance from the center of the plug to the leading edge of the fungus (*x*) and the distance from the edge of the bacterial growth to the growing edge of the fungus (*y*) were measured and the inhibition index (*i*) was calculated as *i* = *y/(x + y)* × 100%. The model biocontrol strain *Pseudomonas protegens* Pf-5 was used as a positive control while negative control plates contained only fungus. All assays were repeated twice with 3–6 replicates per isolate. The most active antagonists were identified by amplifying 16S rRNA with primers 8F and 1492R, end-sequencing the resultant amplicons, and analyzing sequences with the Sequence Match tool of the Ribosomal Database Project (RDP-II)^1^.

### Screening of Soil DNA and Bacterial Isolates for the Presence of Genes Involved in the Production of Phenazines and 2,4-Diacetlphloroglucinol

Samples of DNA extracted from plant rhizosphere or selected bacterial isolates were screened by PCR for the presence of genes involved in the synthesis of phenazines and 2,4-diacetlphloroglucinol (2,4-DAPG), since both of these antimicrobial compounds were implicated in the biological control of phytopathogenic fungi (including *Fusarium* spp.) by certain groups of beneficial rhizobacteria (Weller et al., 2007; Mazurier et al., 2009). The amplifications were performed in 25-μL reaction mixtures containing PCR reagents, 1 μL of template (10-fold diluted soil DNA) and primers targeting the phenazine biosynthesis gene *phzF* (primers Ps_up1 and Ps_low1) (Mavrodi et al., 2010), or the 2,4-DAPG biosynthesis gene *phlD* (primers BPR4 and B2BF) (Gardener et al., 2001). The cycling programs for the amplification of *phzF* and *phlD* loci were as described by Gardener et al. (2001) and Mavrodi et al. (2010), respectively. *Pseudomonas brassicacearum* Q8r1-96 was used as a positive control for the amplification of 2,4-DAPG genes, while *P. synxantha* 2-79 served as a positive control for the presence of phenazine biosynthesis genes.

## Results

### Site Descriptions at the Sampling Time

At the time of sampling in late 2016, field observations showed that all three transects were primarily in *S. alterniflora* dominated intertidal marsh zone. Vegetation percent cover was high, at least 50%, and increased with age of the site. Both *S. alterniflora* and *J. roemerianus* were present at the natural marsh site (control) occupying the mid marsh elevation, but *S. alterniflora* was dominant in contrast to *J. roemerianus* which grew as isolated plants or dense clumps. Sediment was muddy but well bound by extensive root mat, and it was mostly easy to traverse except in unvegetated areas. Vegetation cover was 70–90%, canopy height was typically 60 cm or less and 1.2–1.5 m for *S. alterniflora* and *J. roemerianus*, respectively. During the sampling at the DIMR1 site, we observed some *S. alterniflora* toward an interior lagoon in the low- to mid-marsh zone, but no *J. roemerianus*, which has remained mostly absent for more than 10 years since the original plantings in April 2005. Much of the central part of the transect was in high marsh habitat with sandy sediments that were dominated by *S. patens*. Vegetation percent cover varied from >90 to <10% in sandy areas with vegetation height of less than 60 cm. Other plants observed included *Ipomoea pes-caprae* (bayhops), *Hydrocotyle bonariensis* (largeleaf pennywort), and *D. spicata*. The DIMR2 site was recently planted in the spring of 2016 as part of the ongoing restoration and during sampling had only *S. alterniflora* present in both the low- and mid-marsh elevations. The canopies of the plants were tall for this species ranging between 1.2 and 1.5 m in height, and the vegetation percent cover ranged from 50 to 80% over the site. Sediments were muddy clay, with surface biofilm indicating regular submergence. Most areas were compact enough to walk without difficulty. The analysis of sediments revealed substantial differences between the three sampled areas. Sediment cores collected at the natural marsh had the highest overall levels (although not significantly different from DIMR2) of carbon, nitrogen, TOC, and sulfate (Supplementary Table 1). In contrast, samples from DIMR1 were low on C, N, TOC, ammonium, and sulfate. The DIMR1 sediments also contained a significantly higher amount of sand and less silt and clay compared to the natural marsh and DIMR2.

### Microbial Community Analysis

**FIGURE 2:**
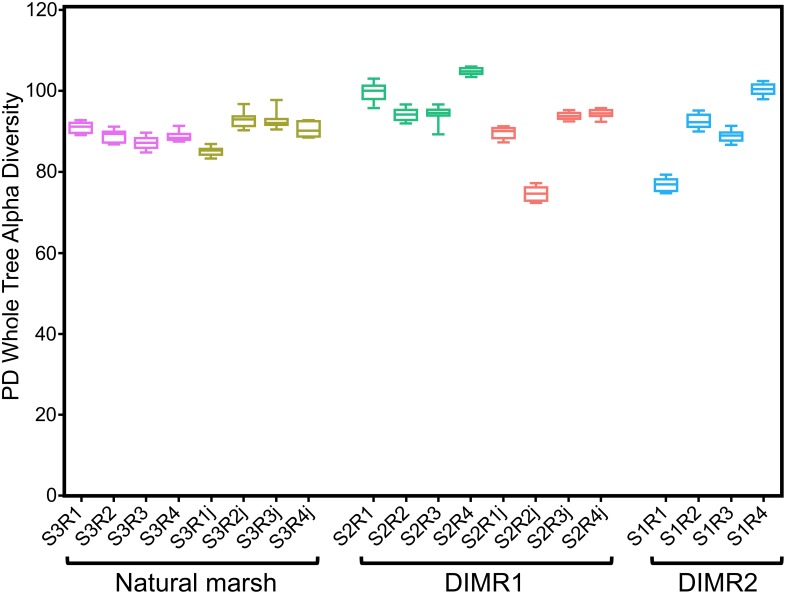
Alpha diversity [Faith’s phylogenetic diversity (PD) whole tree metrics] by plant hosts *Spartina alterniflora* and *Juncus roemerianus* (series labels followed by “j”) at the natural marsh (S3R series) and restored DIMR1 (S2R series) and DIMR2 (S1R series) sites. Samples were rarified to 10,000 reads per sample and compared using a non-parametric *t*-test. No significant differences were found between any of the samples (*P* < 0.5).

**FIGURE 3:**
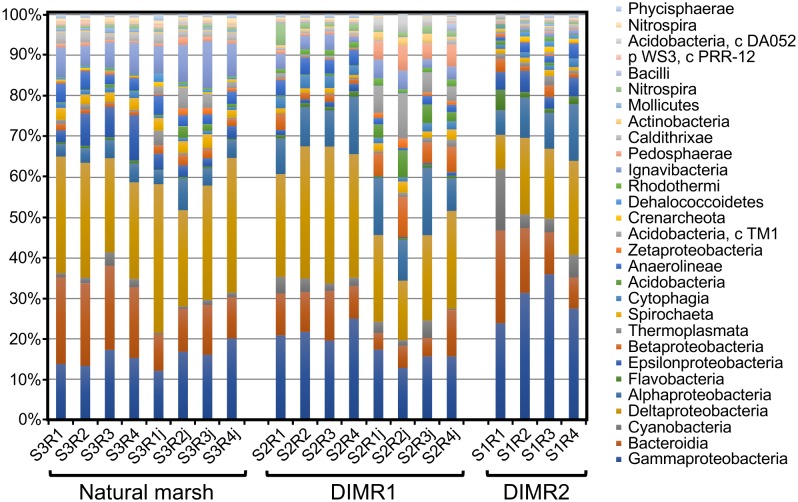
Relative abundance at the class level of the dominant bacterial taxa in microbial communities associated with roots of *S. alterniflora* and *J. roemerianus* (series labels followed by “j”) collected at the natural tidal marsh (S3R series) and restored DIMR1 (S2R series) and DIMR2 (S1R series) sites on Deer Island, MS.

We compared bacterial microbiomes associated with roots of *S. alterniflora* and *J. roemerianus* collected at the three sites. The removal of low-quality reads yielded 5 million sequences spanning the V4 region of the 16S rRNA gene (*n* = 4 per sampled plant species per site) with a median read count per sample of 83,000. The high-quality reads were binned at >97% sequence identity into OTUs which were utilized for subsequent analyses (Supplementary Figure 1). The rarefaction analysis [phylogenetic diversity (PD) whole tree] at a depth of 10,000 reads per sample indicated sufficient sampling depth to represent the OTU richness of the studied microbial communities (data not shown). The alpha diversity calculated using Faith’s PD metric showed that differences in alpha diversity between the two restored sites and the natural marsh were not significant (non-parametric *t*-test; *P* > 0.5) (Figure 2). The core rhizosphere microbiomes of *S. alterniflora* and *J. roemerianus* included members of the phyla Proteobacteria, Bacteroidetes, and Acidobacteria as the most numerous taxa represented by OTU numbers. At the class level, samples were dominated in descending order by Gamma-, Delta-, Alpha-proteobacteria, Bacteroidia, and Ignavibacteria and had a high abundance of rare or poorly defined taxa (Figure 3).

**FIGURE 4:**
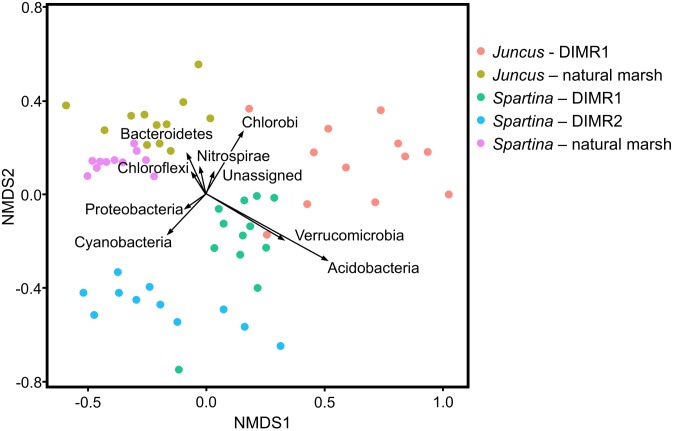
Two-dimensional non-metric multidimensional scaling (NMDS) plot with vectors displaying most abundant bacteria phyla identified in samples from *S. alterniflora* and *J. roemerianus*. The length of the vector is correlated to relative abundance of each of the dominant phyla. Samples are color-coded based on the plant species and sampling site. Controls are natural marsh samples.

Non-metric multidimensional scaling plots based on the Bray–Curtis dissimilarities matrix showed a separation between samples from the natural reference marsh, DIMR1, and DIMR2 (Figure 4). Similar pattern was observed when samples were subjected to the principal coordinate analysis (PCoA) using weighted UniFrac distances (Supplementary Figure 2). These differences in overall microbial community structure between the three sites were significant at a 95% confidence interval. The relative abundance of microbial taxa was then compared at the order level to infer key microbial phylotypes associated with the rhizosphere of plants from the restored and natural marshes. Multivariate analysis identified several taxa that appeared to drive the separation between *S. alterniflora* and *J. roemerianus* and between the natural marsh and restored sites DIMR1 and DIMR2 (Figure 4). Non-parametric multivariate analysis of variance indicated these differences were significant (*F* = 12.34, *P* < 0.001). In particular, Acidobacteria and Verrucomicrobia appeared to be more closely linked with *S. alterniflora* from DIMR1 while Chlorobi seemed to be a driver in the separation of *J. roemerianus* at DIMR1. Furthermore, Cyanobacteria were linked with *Spartina* at DIMR2, whereas Bacteroidetes, Nitrospirae, and Chloroflexi were associated with *Juncus* from the natural marsh (Figure 4).

Differences in the relative abundance of dominant phyla between the sampled communities were also determined at the phylum level using a Kruskal–Wallis test (*P* < 0.001). Overall, Proteobacteria represented a dominant group in all analyzed *Spartina* samples although their levels were significantly higher in both DIMR sites (60% relative abundance) than in the natural marsh (50% relative abundance) (Supplementary Figure 3). In contrast, Bacteriodetes were significantly higher (*P* < 0.001) in the reference marsh with a relative abundance of 20% compared to around only 14% in the two restored sites. All other intersite differences were in agreement with results of the NMDS analysis described above (Supplementary Figure 4). We also used samples collected at the natural marsh and DIMR1 for intrasite comparisons of microbial communities of *Juncus* and *Spartina*. In the reference site, the two plant species had overall similar microbiomes that differed only in the abundance of Bacteriodetes (*P* < 0.001) (Supplementary Figure 5). These results were further supported by NMDS analysis which showed close clustering of samples from the natural marsh regardless of plant species (Figure 4). The examination of communities from DIMR1 revealed significantly higher levels (*P* < 0.001) of Proteobacteria and Bacteriodetes in the rhizosphere of *S. alterniflora*, while Acidobacteria and Verrucomicrobia were more abundant (*P* < 0.001) on roots of *J. roemerianus* (Supplementary Figure 6). The observed differences were most significant with Acidobacteria that were >5-fold higher abundance in *Juncus* as compared to *Spartina*.

### Analysis of Total Culturable Heterotrophic Rhizobacteria in the Rhizosphere of *S. alterniflora* and *J. roemerianus*

The population levels of total culturable heterotrophic bacteria in the rhizosphere of *Spartina* and *Juncus* ranged between log 6.4 and 7.7 CFU g^-1^ root fresh weight, with slightly higher overall populations observed in *S. alterniflora* (Table 2). On ^1^/_3_ KMB, the populations of rhizobacteria were significantly higher on *S. alterniflora* at the DIMR1 site in comparison with DIMR2 and the natural marsh. On ^1^/_10_ TSB, the levels of bacteria recovered from the DIMR1 and DIMR2 were similar but significantly higher than those in the natural marsh. In *J. roemerianus*, the populations of culturable rhizobacteria were highest on ^1^/_3_ KMB in the natural marsh samples, whereas no difference between sites was observed when bacteria were plated on ^1^/_10_ TSB. The actual number of isolates per site ranged from 108 to 190 with a broader range of distinct morphotypes (i.e., colony diameter, shape, elevation, margin, pigmentation, opacity, and texture) observed on ^1^/_10_ TSB in comparison with ^1^/_3_ KMB (Supplementary Figure 7). A total of 751 bacterial isolates were recovered from the rhizosphere of *S. alterniflora*, and *J. roemerianus* collected at the three surveyed Deer Island sites (Table 2). At each site, we observed 20 to 30 different morphotypes, and it is likely that these morphotypes were represented in the final collection by multiple isolates.

**Table 2:** Population densities of culturable aerobic heterotrophic bacteria and the number of isolated bacteria from rhizospheres of *S. alterniflora* and *J. roemerianus* collected on Deer Island.

Site	Plant species	Population density (Log CFU/g^-1^ ± SD)*^a^*		The number of isolates recovered on*^b^*		Total isolates per site
		^1^/_3_ KMB	^1^/_10_ TSB	^1^/_3_ KMB	^1^/_10_ TSB	
DIMR1	*S. alterniflora^c^*	7.5 ± 0.3 A	7.7 ± 0.7 A^∗^	54	98	152
DIMR2	*S. alterniflora*	7.2 ± 0.5 B	7.6 ± 0.6 A^∗^	56	129	185
Natural marsh	*S. alterniflora*	7.0 ± 0.5 B	7.2 ± 0.4 B^∗^	82	108	190
DIMR1	*J. roemerianus^d^*	6.4 ± 0.6 B	6.6 ± 0.7 A†	21	95	116
Natural marsh	*J. roemerianus*	6.7 ± 0.6 A	6.7 ± 0.5 A†	22	86	108

^a^*Population densities of heterotrophic bacteria were determined on ^*1*^/_*3*_ KMB and ^*1*^/_*10*_ TSB by the terminal dilution endpoint assay. *^*b*^*Morphologically distinct rhizobacteria from the roots of *S. alterniflora* and *J. roemerianus* were selected by dilution plating on 1^*1*^/_*3*_ KMB and ^*1*^/_*10*_ TSB. *^*c*^*Differences among treatments with *S. alterniflora* were determined by standard one-way analysis of variance (ANOVA) with mean comparisons among treatments performed by the Fisher’s protected least significant difference (LSD) or ^∗^Kruskal–Wallis tests (both at *P* = 0.05). *^*d*^*Differences among treatments with *J. roemerianus* were determined by the two-sample *t*-test or †Wilcoxon Rank Sum test (both at *P* = 0.05).*

### Plant Growth Promotion and Biocontrol Traits of Bacterial Isolates

Results of the screening indicate that a total of 72 (25.1%) strains were motile with approximately equal distribution between the plant hosts and sites (Table 3). The secretion of siderophores was detected in 100 (34.8%) strains based on their ability to chelate iron from the dye complex and change the color of the indicator O-CAS medium from blue to yellow. The production of siderophores was evaluated using a modified overlay technique that minimized the inhibition of Gram-positive bacteria by hexadecyltrimethylammonium bromide (HDTMA), which is used as an indicator in CAS medium (Perez-Miranda et al., 2007). The production of 1-aminocyclopropane-1-carboxylic acid (ACC) deaminase and IAA by rhizobacteria are important determinants in beneficial plant-bacterial interactions because the release of these compounds results in the formation of longer and more robust root systems (Penrose and Glick, 2003). Our screening identified 10 strains (7.9%) that grew in the defined DF medium supplemented with ACC as a sole nitrogen source, and 21 strains (7.3%) that produced IAA in low (3.9–16.2 fg cell^-1^), medium (32.3–54.3 fg cell^-1^), or high (176.2–215.5 fg cell^-1^) quantities (data not shown). The production of antimicrobial biosurfactants and hydrogen cyanide was detected in 41 (14.3%), and 6 strains, respectively, while the secretion of the protease was noted in a total of 68 (44.7%) strains. Not all isolates grew in culture media used for the detection of ACC-deaminase (DF medium), exoprotease (milk agar), or hydrogen cyanide (TSA medium supplemented with glycine) (Supplementary Figure 8). Hence, the percent of positive strains in each category was calculated based on the number of isolates capable of growing in the corresponding semi-selective or indicator medium. Overall, the frequencies of bacterial isolates with beneficial traits did not differ significantly between the natural marsh and DIMR1 (χ^2^ = 6.9, *P* = 0.3305, d.f. = 6 for *Juncus*, and χ^2^ = 8.05, *P* = 0.0897, d.f. = 4 for *Spartina*). In contrast, we observed a significant difference (χ^2^ = 19.41, *P* = 0.0016, d.f. = 5 for *Spartina*) in the frequencies of beneficial isolates between the natural marsh and recently established DIMR2 site.

**Table 3:** Screening of bacterial isolates from *J. roemerianus* and *S. alterniflora* for traits associated with the competitive rhizosphere colonization and plant growth promotion.

Site and plant species	Plant colonization and growth promotion traits					
	Motility	Biosurfactant	Exoprotease	Siderophores	HCN	IAA	ACC
Natural marsh, *J. roemerianus*	56*^a^* (13)*^b^*	56*^a^* (10)*^b^*	22*^a^* (10)*^b^*	56*^a^* (15)*^b^*	48*^a^* (1)*^b^*	56*^a^* (2)*^b^*	25*^a^*(2)*^b^*
Natural marsh, *S. alterniflora*	61 (19)	61 (8)	38 (19)	61 (20)	53 (0)	61 (0)	18 (0)
DIMR1, *J. roemerianus*	57 (12)	57 (7)	34 (17)	57 (20)	50 (5)	57 (7)	25 (1)
DIMR1, *S. alterniflora*	58 (15)	58 (4)	23 (14)	58 (25)	47 (0)	58 (5)	24 (1)
DIMR2, *S. alterniflora*	55 (13)	55 (12)	35 (8)	55 (20)	54 (0)	55 (7)	35 (6)
Positive strains (%)	25.1	14.3	44.7	34.8	2.4	7.3	7.9

^a^*The number of isolates growing in the indicator medium. *^*b*^*The number of isolates tested positive for the assayed trait.*

### *In vitro* Inhibition of *Fusarium* by Rhizobacterial Strains

Previous studies reported that *S. alterniflora* harbors different species of *Fusarium*, some of which were common in salt marshes affected by SVD (Elmer and Marra, 2011; Elmer, 2014). Among these, *F. palustre* was strongly associated with *S. alterniflora* in the parasite/host relationship manner and was capable of inciting disease in pathogenicity trials. We could not obtain an isolate of *F. palustre* and instead used the plant pathogens *F. pseudograminearum* and *F. culmorum.* Although not identical, the three species are closely related and belong to the same *Fusarium* species complex (i.e., *F. sambucinum*) (Aoki et al., 2014). Screening for the antifungal activity yielded six strains that actively inhibited the mycelial growth of *F. pseudograminearum* and *F. culmorum.* Only one of these strains was isolated from *J. roemerianus* (collected in the natural marsh), while the rest originated from the roots of *S. alterniflora* collected at both DIMR2 and the natural marsh (Table 4). All six strains inhibited *F. pseudograminearum*, but only four out of six antagonized *F. culmorum*. Partial sequencing of 16S rDNA identified strains that were most inhibitory toward *Fusarium* (isolates 461, 449, 577) as members of *Gynuella sunshinyii* (Table 4 and Supplementary Figure 9). Two strains with slightly lower antifungal activity (isolates 9 and 619) were identified as *Bacillus pumilus* that originated from *J. roemerianus* and *S. alterniflora* collected at the natural marsh site. Interestingly, the antagonism toward to *Fusarium* was species-specific, and both *B. pumilus* strains inhibited only *F. pseudograminearum* but not *F. culmorum* (Table 4 and Supplementary Figure 9). The last antagonistic strain, isolate 294, was identified as *Tenacibaculum discolor* and exhibited activity against both species of *Fusarium.* We screened genomic DNA of all antagonistic strains by PCR with primers targeting genes for the synthesis of antifungal metabolites phenazine and 2,4-DAPG. We also used the same PCR assay to screen all samples of soil DNA that were extracted from the rhizosphere of *S. alterniflora* and *J. roemerianus*. The quality of extracted soil DNA was assessed by PCR with universal eubacterial 16S primers 8F and 1492R and revealed that all samples were of high quality and free of PCR inhibitors. The positive controls also worked as expected and produced the 629- and 427-bp amplicons that matched, respectively, the amplified portions of *phlD* and *phzF* genes. In contrast, no amplification of *phlD* or *phzF* occurred in PCR reactions containing rhizosphere soil DNA or DNA extracted from the six strains that actively inhibited *Fusarium* (data not shown).

**Table 4:** Inhibition of *Fusarium pseudograminearum* and *F. culmorum* by strains isolated from the rhizosphere of *S. alterniflora* and *J. roemerianus*^a^.

Strain	Site and plant species	Pathogen inhibition index (% ± SE)*^b^*	
		*F. pseudograminearum*	*F. culmorum*
*Bacillus pumilis* 9	Natural marsh, *J. roemerianus*	5.2 ± 0.8 E	No inhibition
*Tenacibaculum discolor* 294	DIMR2, *S. alterniflora*	19.1 ± 1.0 C	7.4 ± 1.6 C
*Gynuella sunshinyii* 449	DIMR2, *S. alterniflora*	32.1 ± 1.0 B	28.2 ± 1.0 B
*Gynuella sunshinyii* 461	DIMR2, *S. alterniflora*	32.1 ± 1.2 B	27.1 ± 1.1 B
*Gynuella sunshinyii* 577	Natural marsh, *S. alterniflora*	38.8 ± 1.2 A	37.1 ± 1.1 A
*Bacillus pumilis* 619	Natural marsh, *S. alterniflora*	11.9 ± 2.1 D	No inhibition

^a^*Inhibition was assayed on ^*1*^/_*5*_ TSA. *^*b*^*Pathogen inhibition index (*i*) was defined as *i* = *y/(y+x)* × 100, where *x* is the distance from the center of the plug to the leading edge of fungus and *y* is the distance from the edge of the bacterial colony to the growing edge of the fungus. Data are means and standard errors of six replications. Numbers in the same column followed by different letters are significantly different according to Fisher’s protected least significant-difference test (*P* = 0.05).*

## Discussion

To gain a clearer understanding of the sedimentary microbial processes occurring during plant succession after initial restoration, and thereby better inform the potential for long-term stability of restored marsh habitat via beneficial use of dredged material, additional microbial analysis was conducted at Deer Island restoration sites DIMR1 and DIMR2 and compared to the results from a nearby virgin natural marsh. Previously a post-construction assessment showed the site dominated by *S. alterniflora*, *S. patens, D. spicata*, and *J. roemerianus* with no significant differences in plant abundance or diversity values between the reference site and the DIMR1 restored site (Lang, 2012). Similarly, in this study analysis of rhizosphere associated microbial communities for *S. alterniflora* and *J. roemerianus* from the natural and two different aged restored sites showed that the two restoration sites (DIMR1 and DIMR2) had similar soil microbial communities when compared to the natural marsh. Collectively, these findings suggest that native rhizosphere-associated microbial communities established on *S. alterniflora* planted at the restored areas over a short period of time (within 6 month of planting).

Natural tidal marshes are highly productive coastal ecosystems characterized by sediments rich in organic matter resulting from the decay of plant material and root exudation (Bertness, 2006). Because of the frequent waterlogging, the abundant residual organic matter is actively decomposed via anaerobic microbial respiration through sulfate reduction. The sulfate reducers produce hydrogen sulfide and other reduced sulfur compounds that serve as electron donors for sulfur-oxidizing microorganisms, which complete the sulfur cycle (Wasmund et al., 2017). All rhizosphere samples collected across the three study sites showed an abundance of sulfate-reducing and sulfur-oxidizing organisms, which supports the importance of sulfur cycling and is consistent with the anaerobic nature of saturated tidal marsh sediments. The sulfate-reducing group was dominated by Desulfobacterales, which are metabolically flexible and especially well-adapted to tidal marshes due to the ability to tolerate oxidative stress from oxygen penetration in shallow sediments (Wasmund et al., 2017). We also identified the presence of Nitrospirae, which include the thermophilic sulfate-reducing bacteria of the genus *Thermodesulfovibrio*. Analysis of the community data showed that members of the family *Thermodesulfovibrionaceae* were present but represented <1% total relative abundance and differences in abundance between restored and control sites were not statistically significant (*P* < 0.05). The most abundant group of sulfur oxidizers was represented by Chromatiales that use sulfides as a reducing agent, which is further oxidized to elemental sulfur. The roots of *Spartina* and *Juncus* also supported diverse Bacteroidia, which are known to carry multiple sulfatase genes and specialize in degrading sulfated organics in anoxic subsurface sediments (Baker et al., 2015).

The presence and oxidation state of sulfur can influence vegetation patterns and contribute to the natural zonation of wetland plant species (Lamers et al., 2013). In the sites studied, *S. alterniflora* is typically the dominant species in the low- to mid-marsh zone, where abiotic stress including inundation and anaerobic soil conditions can lead to high sulfide concentrations. Sulfide is toxic to plant metabolism, and it is thought that marsh grasses benefit from the interaction with sulfur-oxidizing microorganisms that help to lower hydrogen sulfide levels in the rhizosphere. Results of the intersite comparisons on Deer Island identified Bacteroidetes and Nitrospirae among taxa associated with *J. roemerianus* from the natural marsh, while Chlorobi (a group known to contain sulfur-oxidizers) were a driver in the separation of *Juncus* microbiome at DIMR1. Such fluctuations in the abundance of taxa involved in the transformation of different sulfur compounds may be of importance for the plant fitness and ecosystem functioning in newly created marshes. *Juncus roemerianus*, which typically dominates in mid-marsh elevations in the northern Gulf of Mexico, was frequently found to occur in low abundance (DIMR1) or be entirely absent (DIMR2) from this zone in the two restored sites, potentially related to site-specific sediment microbial processes including sulfate reduction. Other significant differences in the microbial communities were observed between the rhizospheres of *S. alterniflora* and *J. roemerianus.* Proteobacteria and Bacteriodetes were significantly higher (*P* < 0.001) in samples recovered from roots of *Spartina* while Acidobacteria and Verrucomicrobia were significantly higher (*P* < 0.001) in the rhizosphere of *Juncus*. The difference was greatest with Acidobacteria which were fivefold higher in *Juncus* dominated areas. Although the significance of these differences for the establishment of restored marshes is unclear, both Acidobacteria and Verrucomicrobia represent diverse lineages of bacteria that are particularly abundant in soil habitats and are involved in the subsurface cycling of carbon and nitrogen (Schlesner et al., 2006; Kielak et al., 2016). Previously, an analysis of vegetated sediments in Louisiana saltmarshes indicated that *S. alterniflora* microbial sediment communities had greater proportions of Bacteroidetes and Lentisphaerae compared to *J. roemerianus*, while *J. roemerianus* sediments contained higher proportions of α-Proteobacteria and β-Proteobacteria (Rietl et al., 2016). The predominance of Proteobacteria and Bacteriodetes in some of our samples is consistent with results of that study.

It is likely that the observed intersite variations in the composition of rhizosphere microbial communities are partially driven by differences in the properties of the dredged material that was used for the establishment of artificial marshes at the two DIMR sites. Results of our analyses revealed that sediments from DIMR2 had more silt and clay and more closely resembled samples from the natural marsh (Supplementary Table 1). In contrast, sediments from DIMR1 contained significantly less sulfate, nitrate, and organic matter, and had higher amounts of sand as a result of regular rebuilding of the berm area to replace losses from ongoing erosion. The coarse sediments at DIMR1 are probably more susceptible to oxygen exposure and tidal flushing of porewater nutrients in comparison to finer sediments at DIMR2 and the reference site. Higher levels of dissolved O_2_ and redox potentials were previously reported in restored marshes constructed with sandy sediments (Craft et al., 1991).

Plants have developed various biochemical and physiological mechanisms to respond and adapt to stress conditions. One of them is the fostering of root-associated microbial communities that positively influence plant fitness in response to abiotic stressors (e.g., drought, salt, temperature, and soil pollution) (Dimkpa et al., 2009). The capacity of rhizobacteria to alleviate the detrimental effect of abiotic stress is a multifactorial phenotype. Beneficial rhizobacteria improve the nutritional status of the host plant by fixing atmospheric nitrogen, solubilizing inorganic phosphate, and secreting iron-chelating siderophores (Kim et al., 2011). Many root-colonizing microorganisms stimulate plant organ development through the production of phytohormones such as auxins, cytokinins or gibberellins, which stimulate the formation of root hairs, root growth, and branching, and can improve mineral and nutrient uptake (Kim et al., 2011). Certain beneficial rhizobacteria are capable of modulating levels of the plant hormone ethylene, which leads to the formation of longer roots and enhanced growth following environmental stress. Such bacteria produce the enzyme 1-aminocyclopropane-1-carboxylate (ACC) deaminase, which removes amine group from the immediate precursor of ethylene (Gamalero and Glick, 2015). In comparison to terrestrial plants, the diversity of plant growth-promoting rhizobacteria in tidal marshes and their effect on plant health remains poorly understood. In this study, we surveyed roots of *S. alterniflora* and *J. roemerianus* for culturable rhizobacteria with traits that are commonly associated with competitive rhizosphere colonization and plant growth promotion. Our results demonstrated the presence of bacteria capable of secreting iron-chelating siderophores, antimicrobial metabolites (exoprotease, biosurfactants, and HCN), and the plant growth-promoting metabolites ACC deaminase and IAA (Table 3). The 16S rRNA-based identification of selected isolates revealed that these organisms were taxonomically diverse and included Gram-positive Firmicutes (*Bacillus*), as well as Gram-negative Flavobacteria (*Tenacibaculum*), Alphaproteobacteria (*Marimonas*), and Gammaproteobacteria (*Gallaecimonas, Gynuella, Halomonas, Photobacterium, Vibrio*) (Supplementary Table 2). Although the 16S based community analysis revealed that all these taxa were present at the on relative abundance of <1% (*Vibrio* spp. were the most abundant at 0.8%), their population levels estimated by dilution plating were between 10^6^ and 10^7^ CFU g^-1^ root fresh weight. These values are on par with population levels reported for some well-characterized groups of beneficial rhizobacteria in terrestrial agroecosystems (Weller et al., 2007; Kinkel et al., 2012). Some taxa characterized in this study were also recovered previously from small cordgrass (*Spartina maritima*) and denseflower cordgrass (*Spartina densiflora*) collected in salt marshes of the south Atlantic Spanish coast (Mateos-Naranjo et al., 2015; Paredes-Paliz et al., 2016), and treatment of *Spartina* with these bacteria improved plant growth in soil polluted with heavy metals (Mesa et al., 2015). Interestingly, the overall frequencies of isolates with beneficial traits differed between *Spartina* from the natural marsh and the recently established DIMR2, potentially suggesting that the natural disturbance may affect the dynamics of beneficial microorganisms in the rhizosphere of marsh plants.

Previous culture-based and culture-independent surveys of *S. alterniflora* and *J. roemerianus* from tidal marshes revealed the presence of diverse fungal communities (Gillevet et al., 2009; Walker and Campbell, 2010; Neori and Agami, 2017). While most of these fungi are considered harmless endophytes, surface saprophytes or even mutualistic symbionts (mycorrhizae), some species can act as opportunistic pathogens when host plants are stressed by the environment. Under certain conditions, populations of *S. alterniflora* and *J. roemerianus* in the Gulf Coast marshes undergo a rapid and progressive decline known as saltwater marsh dieback (SMD). One of the most severe SMD events occurred in 2000 and affected 100,000 hectares of salt marsh along Louisiana’s coast (Elmer et al., 2013). Interactive climate conditions and sea level anomalies are thought to be the primary cause of the sudden marsh dieback. Biotic factors associated with SMD are poorly understood, but independent assessments of the possible role of pathogens during dieback events in Louisiana and New England suggested the involvement of soilborne fungi of the genus *Fusarium* (Elmer and Marra, 2011). It has been proposed that *Fusarium* normally has an endophytic association with *Spartina* but may turn pathogenic in plants predisposed by abiotic stressors. We tested a subset of bacterial isolates from the rhizosphere of *S. alterniflora* and *J. roemerianus* for their capacity to inhibit plant pathogenic fungi *F. pseudograminearum* and *F. culmorum.* Our screening identified several Gram-positive and Gram-negative organisms that effectively antagonized one or both species of *Fusarium*, with the best antifungal activity observed in strains 441, 461, and 577 of *G. sunshinyii* (Table 4). Interestingly, *Gynuella* is a poorly characterized halophilic bacterium that was recently discovered in the rhizosphere of a salt-marsh sedge (*Carex scabrifolia*) and proved to inhibit a broad range of plant pathogenic fungi and oomycetes, including *Botrytis, Colletotrichum, Pythium, Rhizoctonia*, *Sclerotinia*, and *Phytophthora* (Chung et al., 2015). In terrestrial ecosystems, beneficial root-colonizing (rhizosphere) bacteria play a significant role in defending plants against soilborne pathogens (Mendes et al., 2011; Philippot et al., 2013; Raaijmakers and Mazzola, 2016). The suppression of pathogens by beneficial rhizobacteria is a complex phenomenon and often results from the synergistic interaction of different types of biocontrol mechanisms. Most often the biocontrol results from antagonism, which involves the destruction of the pathogen by antibiotics, toxins, and lytic enzymes, or parasitism/predation of the pathogen by the biocontrol agent. Alternatively, antagonism may result from indirect interactions where the beneficial microorganism suppresses the pathogen by competing for essential nutrients or space. It is tempting to speculate that species like *Gynuella* represent a specific group of beneficial microorganisms that are associated with roots of marsh plants and may contribute to their protection against different fungal pathogens. As a follow up to this study, we sequenced genomes of *G. sunshinyii* 449, *G. sunshinyii* 577, *M. ostereistagnii* 398, and *M. spartinae* 468 and are currently in the process of analyzing them for the potential plant growth promotion and antibiotic biosynthesis genes.

The analysis of recently sequenced microbial genomes has revealed that most beneficial rhizobacteria can produce multiple antibiotics and antagonistic metabolites, often with broad and overlapping spectra of activity. To date, however, only a small proportion of such antibiotics have been studied, as is evidenced by the recent flood of genomic-based discoveries of new antimicrobial and insecticidal compounds. In biocontrol strains, the biochemistry and genetics of antibiotic production are best studied in members of *Bacillus, Streptomyces*, and *Pseudomonas* (Gross and Loper, 2009; Raaijmakers et al., 2010; Barka et al., 2016). The types of antibiotics synthesized by these biocontrol agents are very diverse and include peptides and polyketides and hybrids thereof, cyclic lipopeptides, derivatives of chorismate and amino acids, macrolides and aminoglycosides. In terrestrial agroecosystems, rhizosphere-dwelling bacteria that produce 2,4-diacetylphloroglucinol, phenazines, and various polyketides have been implicated as key antagonistic components of microbial communities suppressive to diseases caused by *Fusarium* and other phytopathogenic fungi (Weller et al., 2007; Mazurier et al., 2009; Mendes et al., 2011; Kinkel et al., 2012; Cha et al., 2016). We screened all bacterial isolates that antagonized *Fusarium*, as well as samples of soil DNA extracted from the rhizosphere of *S. alterniflora* and *J. roemerianus* by PCR targeting genes for the synthesis of 2,4-diacetylphloroglucinol and phenazines. Results of these screens were negative suggesting that the antagonism was likely due to the presence of other types of antimicrobials.

It can take decades for sedimentary cycles in restored marshes to approach reference condition and the contribution of the sediment microbial communities to these processes is still poorly elucidated. In this study, we addressed this gap by comparing rhizosphere microbiomes of *S. alterniflora* and *J. roemerianus* from two restored tidal marshes and the natural reference marsh located at Deer Island, MS. Our results revealed that plants from the restored and reference areas supported similar microbial diversity indicating the rapid colonization of planted grasses with indigenous soil microbiota. Although close in composition, the microbial communities from the three studied sites differed significantly in the relative abundance of specific taxa. The observed differences are likely driven by the host plant identity and properties of sediment material used for the creation of restored marshes. Some of the differentially distributed groups of bacteria include taxa involved in the cycling of carbon, nitrogen, and sulfur, and may influence the succession of vegetation at the restored sited to climax condition. We also demonstrated that plants from the restored and reference sites vary in the frequency of culturable rhizobacteria that exhibit traits commonly associated with the promotion of plant growth and suppression of phytopathogenic fungi. Our findings will contribute to the establishment of benchmarks for the assessment of the outcome of coastal restoration projects in the Gulf of Mexico and better define factors that affect the long-term resiliency of tidal marshes and their vulnerability to climate change.

## Author Contributions

DM, OM, PB, and KI conceived the research project. OM, DM, SH, and PB collected field samples. OM, SN, and SH extracted soil DNA and isolated and characterized culturable rhizobacteria. CJ and JE performed Illumina sequencing and conducted the microbiome analysis. DM, OM, CJ, JE, PB, and KI wrote the manuscript. All authors contributed to the manuscript revision.

## Conflict of Interest Statement

The authors declare that the research was conducted in the absence of any commercial or financial relationships that could be construed as a potential conflict of interest.

## Footnotes

1 https://rdp.cme.msu.edu/
