# Supplementary material for: Rhizosphere Microbial Communities of Spartina alterniflora and Juncus roemerianus From Restored and Natural Tidal Marshes on Deer Island, Mississippi
Source: Front Microbiol. 2018 Dec 11;9:3049. doi: 10.3389/fmicb.2018.03049 (PMC6297177; doi:10.3389/fmicb.2018.03049)
Supplement: Supplementary file 1 [file Data_Sheet_1.PDF]

Supplementary Table 1. Analysis of sediments collected at DIMR1, DIMR2, and natural marsh.

Site	NH_4 (mg/kg)	SO_4 (g/kg)	TOC (%)	C (%)	N (%)	Sand (%)	Silt (%)	Clay (%)
DIMR1	3.0 ± 0.4 a	1.2 ± 0.4 b	0.3 ± 0.1 b	0.4 ± 0.1 b	0.02 ± 0.01 b	86.0 ± 6.6 a	6.3 ± 2.5 b	7.7 ± 4.2 b
DIMR2	11.9 ± 4.4 a	3.5 ± 0.8 ab	2.2 ± 0.7 ab	2.5 ± 0.7 ab	0.13 ± 0.04 ab	49.4 ± 10.4 b	24.3 ± 4.6 a	26.3 ± 5.9 a
Natural marsh	10.6 ± 2.5 a	4.7 ± 0.9 a	3.2 ± 0.9 a	3.8 ± 0.9 a	0.18 ± 0.05 a	53.4 ± 3.7 b	26.1 ± 3.5 a	20.6 ± 0.9 ab

Differences among treatments were determined by One-Way Analysis of Variance (ANOVA). Numbers in the same column followed by different letters are significantly different according to Fisher's protected least significant-difference test ($P = 0.05$).

Supplementary Table 2. Taxonomic identity of selected rhizosphere isolates exhibiting traits associated with the competitive colonization and growth promotion of the host plant.

Strain[b]	Source	Plant colonization and growth promotion traits[a]						
		Biosurfactant	Motility	Exoprotease	Siderophore	HCN	ACC	IAA
Bacillus aquamaris 368	Natural marsh, *S. alterniflora*	-	+	-/+	+/+	-/+	-/-	-/+
Bacillus pumilis 9	Natural marsh, *J. roemerianus*	+	-	+/+	-/+	-/+	-/+	-/+
Bacillus pumilis 120	Natural marsh, *S. alterniflora*	+	+	+/+	+/+	-/+	-/-	-/+
Bacillus pumilis 619	Natural marsh, *S. alterniflora*	+	-	-/+	-/+	-/+	-/-	-/+
Bacillus pumilis 633	Natural marsh, *S. alterniflora*	+	+	-/+	-/+	-/+	-/-	-/+
Bacillus pumilis 648	Natural marsh, *S. alterniflora*	+	+	+/+	-/+	-/+	-/-	-/+
Gallaecimonas pentaromativorans 681	DIMR1, *S. alterniflora*	-	-	+/+	+/+	-/+	-/+	-/+
Gynuella sunshinyii 577	Natural marsh, *S. alterniflora*	+	-	+/+	+/+	-/+	-/+	-/+
Gynuella sunshinyii 449	DIMR2, *S. alterniflora*	+	-	+/+	+/+	-/+	-/-	-/+
Gynuella sunshinyii 461	DIMR2, *S. alterniflora*	+	-	+/+	+/+	-/+	-/-	-/+
Halomonas taenensis 143	DIMR1, *S. alterniflora*	+	-	-/-	+/+	-/+	-/-	+/+
Marimonas ostreistagni 398	DIMR1, *J. roemerianus*	-	+	-/-	-/+	-/+	-/-	+/+
Marimonas spartinae 39	DIMR2, *S. alterniflora*	+	-	-/+	+/+	-/+	+/+	+/+
Marimonas spartinae 44	DIMR2, *S. alterniflora*	+	-	-/+	+/+	-/+	-/-	+/+
Marimonas spartinae 468	DIMR2, *S. alterniflora*	+	-	-/+	+/+	-/+	-+	+/+
Photobacterium ganghwense 712	Natural marsh, *S. alterniflora*	-	-	-/-	+/+	+/+	-/-	-/+
Pseudoalteromonas elyakovii 776	Natural marsh, *S. alterniflora*	-	-	+/+	+/+	+/+	-/-	-/+
Pseudoalteromonas elyakovii 781	DIMR1, *J. roemerianus*	-	-	+/+	+/+	+/+	-/-	-/+
Tenacibaculum discolor 294	DIMR2, *S. alterniflora*	-	-	-/+	+/+	-/+	-/+	-/+
Vibrio fluvialis 373	Natural marsh, *S. alterniflora*	-	-	-/+	+/+	-/+	-/-	-/+
Vibrio maritimus 675	DIMR1, *S. alterniflora*	-	-	+/+	+/+	-/+	-/+	-/+
Vibrio maritimus 687	DIMR1, *S. alterniflora*	+	-	+/+	+/+	-/+	-/+	+/+
Vibrio sinaloensis 709	DIMR1, *J. roemerianus*	-	-	+/+	+/+	-/+	-/-	+/+
Vibrio sinaloensis 715	DIMR1, *J. roemerianus*	-	-	-/+	-/+	-/+	-/+	+/+
Vibrio sinaloensis 800	DIMR1, *J. roemerianus*	-	-	+/+	+/+	-/+	-/+	+/+
Vibrio sinaloensis 803	DIMR1, *J. roemerianus*	-	-	+/+	+/+	-/+	-/+	-/+
Vibrio sinaloensis 671	DIMR1, *S. alterniflora*	-	-	+/+	+/+	-/+	-/+	-/+
Vibrio sinaloensis 673	DIMR1, *S. alterniflora*	+	-	+/+	+/+	-/+	-/+	+/+
Vibrio sinaloensis 680	DIMR1, *S. alterniflora*	-	-	+/+	+/+	-/+	-/+	+/+
Vibrio sinaloensis 683	DIMR1, *S. alterniflora*	-	-	+/+	+/+	-/+	-/+	+/+

[a] +/+ - the isolate is positive for the tested trait and grows on the indicator medium; -/+ - the isolate is negative for the tested trait but grows on the indicator medium; -/- - the isolate does not grow on the indicator medium. [b]Isolates were identified by end-sequencing of 16S rRNA amplicons followed by the analysis with the Sequence Match tool of the Ribosomal Database Project (RDP-II) (https://rdp.cme.msu.edu/).

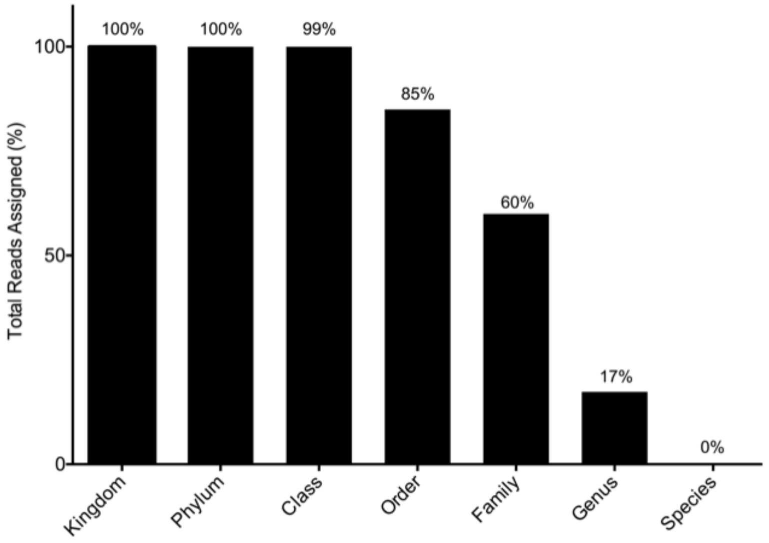

Supplementary Figure 1. Proportion of total reads assigned to different taxonomic ranks.

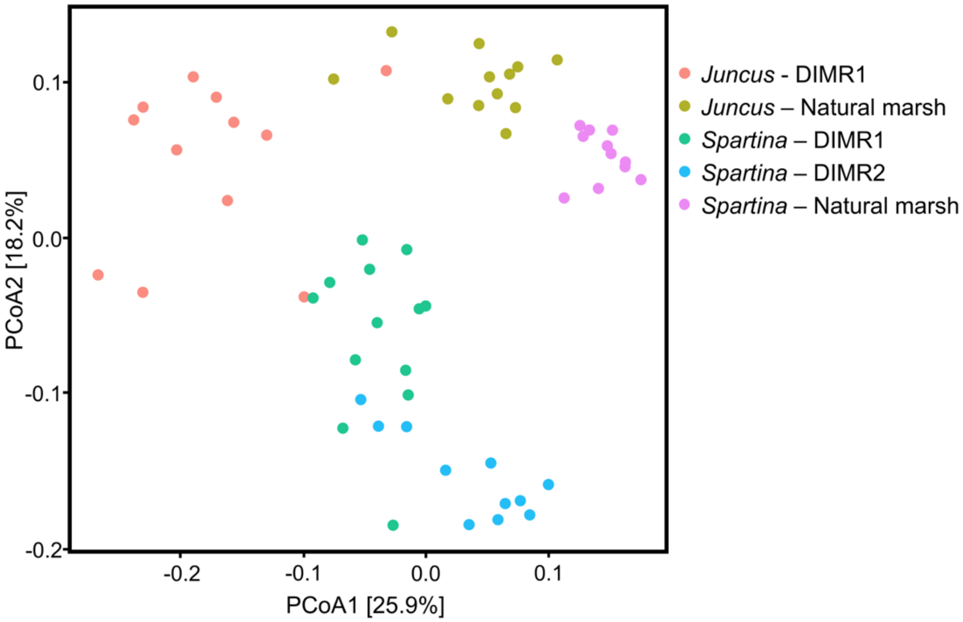

Supplementary Figure 2. Comparison of microbial communities associated with *S. alterniflora* and *J. roemerianus* at the natural and restored tidal marshes on Deer Island, MS. Weighted UniFrac distance PCoA plots comparing three principal components are shown. Control samples are the natural marsh, *Juncus* 05 and *Spartina* 05 are from site DIMR1 while *Spartina* 16 samples are from DIMR2.

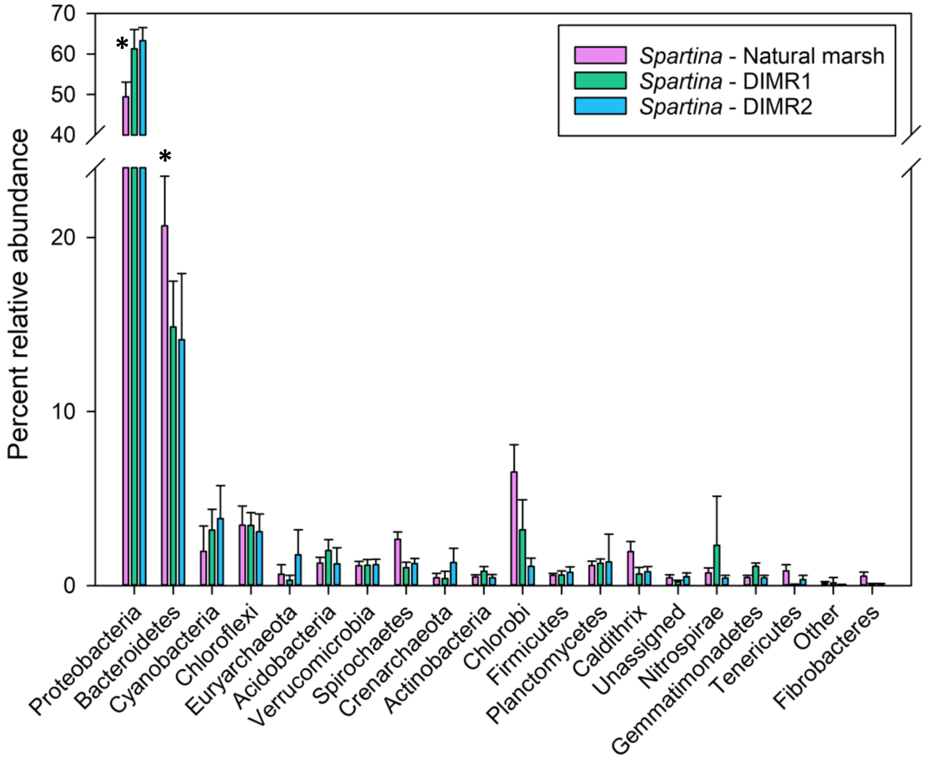

Supplementary Figure 3. Comparison of the relative abundance of major phyla associated with *Spartina* at the natural and restored marsh sites. Asterisks indicate significant differences according to Kruskal-Wallis test ($P < 0.001$).

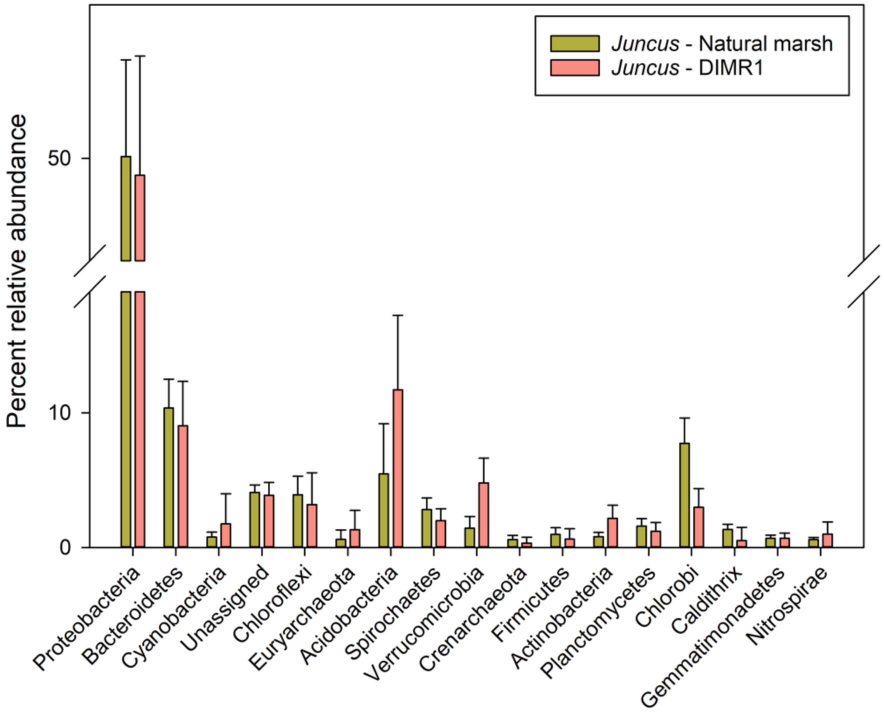

Supplementary Figure 4. Comparison of the relative abundance of major phyla associated with *Juncus* at the natural and restored marsh sites.

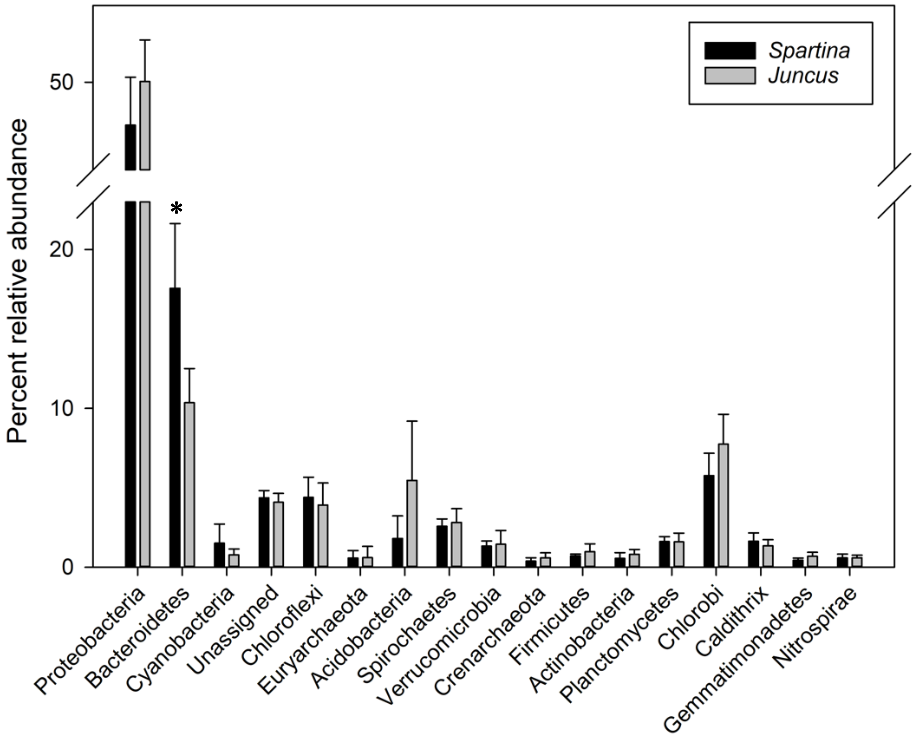

Supplementary Figure 5. Comparison of the relative abundance of major phyla associated with *Juncus* at the natural marsh site. Asterisks indicate significant differences according to Kruskal-Wallis test ($P < 0.001$).

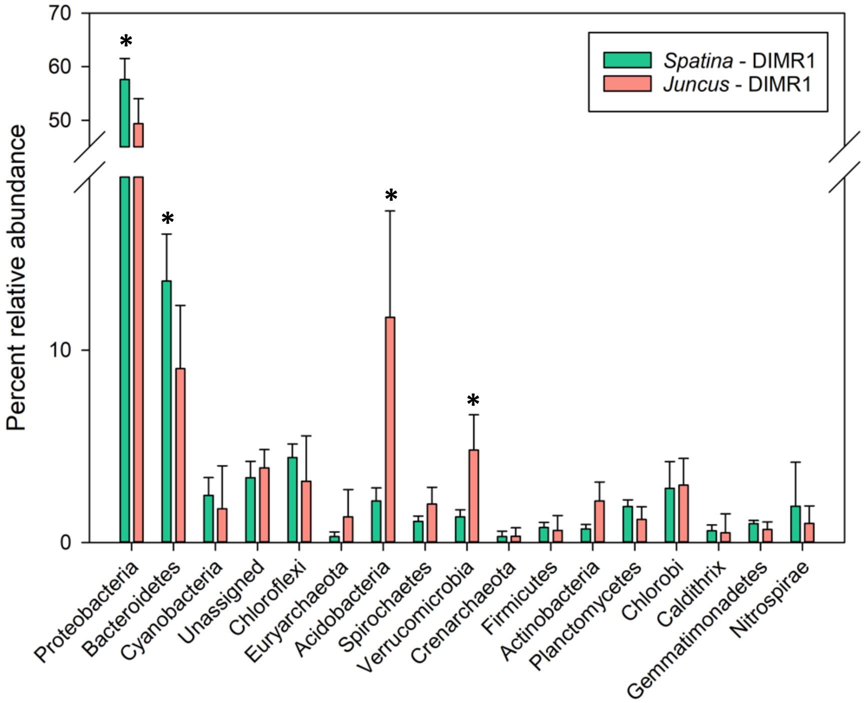

Supplementary Figure 6. Comparison of the relative abundance of major phyla associated with *Spartina* and *Juncus* at the DIMR1 site. Asterisks indicate significant differences according to Kruskal-Wallis test ($P < 0.001$).

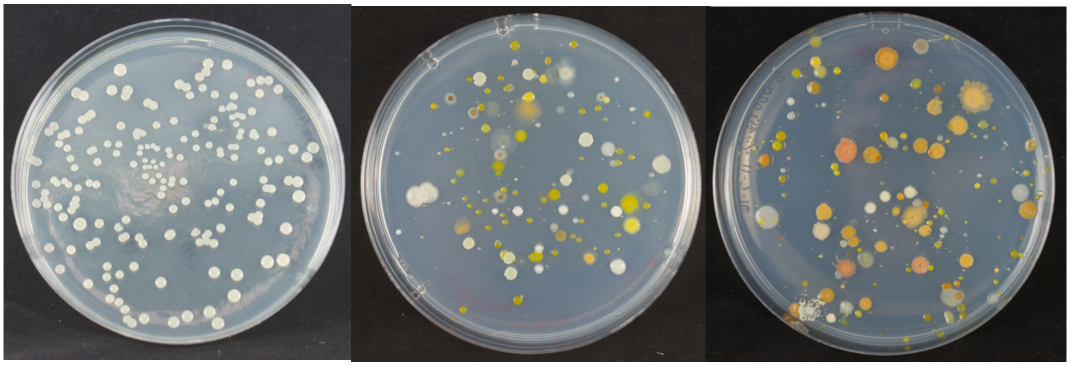

Supplementary Figure 7. Examples of bacterial morphotypes recovered from the rhizosphere of *S. alterniflora* on $^{1}/_{3}$ KMB (left panel) and $^{1}/_{10}$ TSB (center and right panels).

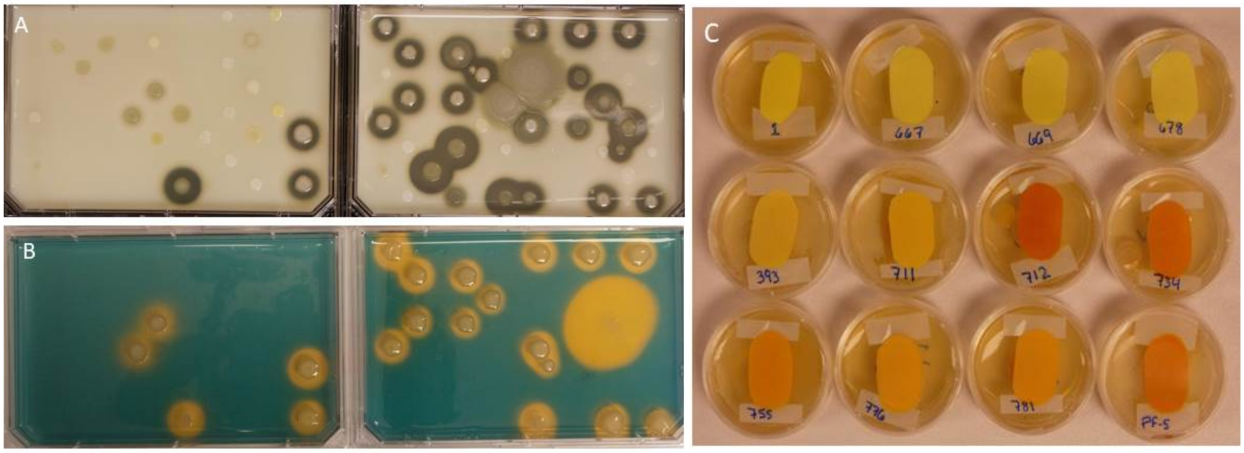

Supplementary Figure 8. Production of exoprotease (A), siderophores (B) and hydrogen cyanide (C) by bacteria isolated from the rhizosphere of *S. alterniflora* and *J. roemerianus.* Panel A – the clearing zone surrounding bacterial growth on skim milk agar is indicative of the exoprotease production. Panel B – presence of a yellow halo around bacterial growth in indicative of siderophore production. Panel C – the change of color on the indicator paper from yellow to bright orange indicates the generation of hydrogen cyanide.

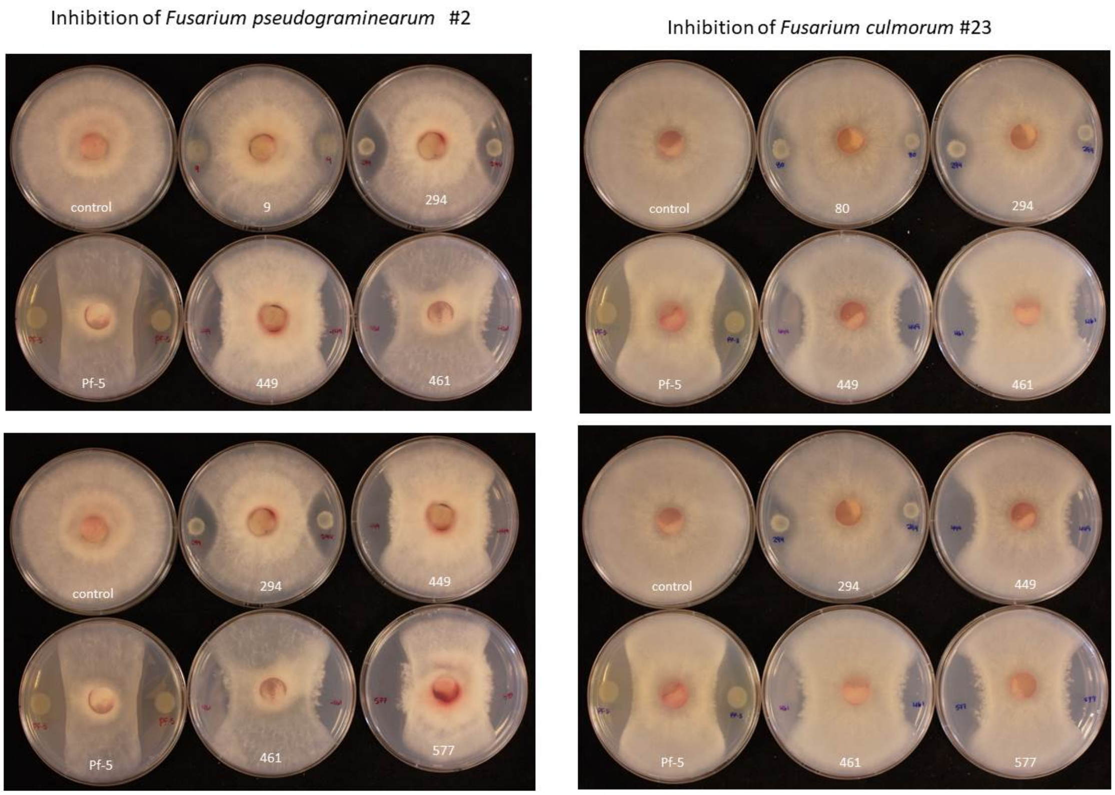

Supplementary Figure 9. Inhibition of plant pathogens *Fusarium pseudograminearum* #2 and *Fusarium pseudograminearum* #23 by selected bacteria isolated from the rhizosphere of *S. alterniflora* and *J. roemerianus.*
